# TAG (Tube and Graft) Sandwich Technique: A Novel Single-Stage Scleral Reinforcement and Aqueous Drainage Tube Implantation

**DOI:** 10.1155/2021/6698919

**Published:** 2021-07-14

**Authors:** Faisal Ahmed, Nada G. Mohamed

**Affiliations:** ^1^The Western Eye Hospital, Imperial College Healthcare NHS Trust, 153-173 Marylebone Rd, London, UK; ^2^The Imperial College Ophthalmic Research Group (ICORG), Imperial College London, 153-173 Marylebone Rd, London, UK; ^3^The Ophthalmology Department, Faculty of Medicine, Alexandria University, Champollion Street, Alexandria, Egypt; ^4^Whipps Cross Hospital, Barts Health NHS Trust, Whipps Cross Rd, Leytonstone, London, UK

## Abstract

**Purpose:**

Refractory glaucoma patients continue to require surgical intervention in the form of trabeculectomy surgery or glaucoma drainage device (GDD). Those patients that require a GDD but have thin sclera or scleromalacia present a challenge.

**Methods:**

In this article, we present a novel “TAG sandwich” single surgical procedure in which thinned sclera is reinforced with a pericardial patch graft (“bottom layer of the sandwich”) allowing safe implantation of the GDD (“the tube sandwich filling”) and then placing another patch graft on top of the tube part of the GDD (“top layer of the sandwich”). The surgery was performed on an open-angle glaucoma patient with a generalized thin sclera and uncontrolled intraocular pressure despite maximal topical medication and oral acetazolamide.

**Results:**

Reinforcing a compromised sclera with a pericardium patch graft allowed the safe implantation of a glaucoma drainage device. The patient's intraocular pressure was safely controlled at 7 mmHg almost 1-year postsurgery without intraocular pressure-lowering drops.

**Conclusions:**

This scleral strengthening procedure can be considered by readers in other ocular surgeries where there is a risk of scleral perforation, as well as part of a combined surgery where refractory glaucoma patients with thin sclera require scleral reinforcement to allow for safer implantation of a glaucoma drainage device.

## 1. Introduction

Glaucoma drainage devices (GDD) are increasingly utilized with a survey in the US reporting an increase from 7,788 in 2003 to 12,021 in 2012 (1). GDDs are used in patients with advanced refractory glaucoma (2, 3). In cases with thin sclera or anterior staphyloma, trabeculectomy surgery can be difficult with possible scleral melting; perforated flaps, and surgical failure (4). In these cases, implantation of a glaucoma drainage device (GDD) can be considered (2, 3). However, this does come with additional risk in patients who have preexisting thin sclera.

The senior surgeon FA had already described a 2-stage scleral reinforcement with GDD implant, with the thin sclera reinforced in the first operation, and 1 month later, a GDD was implanted (5).

This short article describes a novel TAG sandwich technique to strengthen scleral thinning and implant glaucoma drainage device in a single-staged operation. Pericardial tutoplast (Innovative Ophthalmic Products, Costa Mesa, California, USA) was used for scleral reinforcement of thin sclera (“bottom layer of sandwich”) followed by implantation of a GDD on top of the scleral reinforcement (“middle tube layer of sandwich”) followed by gluing of another pericardial Tutoplast^Tm^ graft on top of the tube (“top layer of sandwich”), in a complex case of refractory primary open-angle glaucoma with generalized thin sclera.

## 2. Case Report

Our patient was a 29-year-old lady with Ehler-Danlos syndrome, with uncontrolled advanced open-angle glaucoma. She had previously lost vision in the right eye after an unsuccessful retinal detachment repair. Her only seeing left eye had thinned blue sclera which was considered to be an ocular manifestation of Ehler-Danlos syndrome (6).

Other ocular features that were present in our case included bilateral microcornea, which was reported with Ehler-Danlos type VI but to a lesser extent than Keratoglobus (7). The patient also has pathological myopia which is also seen in Ehler-Danlos patients (8).

Despite maximum medical therapy, and attempted micropulse cyclophotocoagulation (to avoid intraocular surgery), her intraocular pressure remained uncontrolled. Her IOP maintained at 26 mmHg on full topical intraocular pressure-lowering drops, in addition to oral: acetazolamide 250 mg three times a day, and unfortunately, the patient was symptomatic with deteriorating visual fields and vision.

The patient's axial length in this eye was 29.84 mm, and horizontal corneal diameter as shown by white-to-white measurement was 9.83 mm. Her visual acuity preoperatively was 6/36, with a refraction of -11.75 sphere and -1.00 cylinder axis 95 degrees. Her central corneal thickness was 443 microns (thin cornea), and she had grade 4 on Van Herick and AC depth of 3.04 mm as evident from her biometry.

Other Ehler-Danlos-related systemic manifestations in our patient included mitral valve prolapse, which reduced her ASA (American Society of Anesthesiologists classification) rating. The decision was made to implant a glaucoma drainage device. However, scleral reinforcement was required as generalized blue sclera made tube implant challenging.

Although the senior author FA had already described a two-step procedure of scleral reinforcement with pericardial Tutoplast^Tm^ surgery initially with glaucoma tube implant as a second procedure 1 month later, this would have meant 2 anesthetics and delay in IOP control (5). Therefore, it was decided to perform a one surgery scleral strengthening and tube surgery—using the “TAG sandwich technique” under general anesthetic.

**Figure 1:**
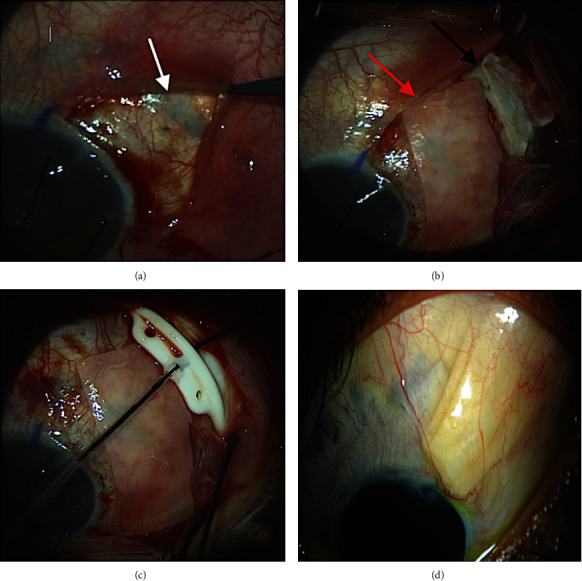
(a) shows scleral thinning evident after conjunctival dissection (white arrow). (b) shows Tutoplast^Tm^ in place covering the bed of the plate (black arrow) and the tube (red arrow). (c) shows the plate and the tube in place over the Tutoplast^Tm^. (d) shows postoperative slit-lamp picture of the tube in place with over lying Tutoplast^Tm^.

The first part of the procedure consisted of a superior peritomy, a 7/0 silk corneal traction suture was used to enhance visualization, and care on taking the suture was made to allow enough depth and not too much traction to avoid cheese wiring of the suture. Reinforcement of the existing thin sclera (“bottom layer of sandwich”) from the limbus with a double layer of pericardial Tutoplast^Tm^ was then performed, and the Tutoplast^Tm^ was glued onto the sclera with Tisseel fibrin sealant (Baxter AG, Vienna, Austria). The sclera under the plate was also reinforced by gluing a single layer of pericardial Tutoplast^Tm^ to it as a precaution. The second part of the procedure was to place a (nonvalved) Baerveldt^R^ BG 101-250 (Abbot Medical Optics, Santa Ana, California, USA) GDD over the Tutoplast^Tm (^“middle tube layer of sandwich”), and the plate was then sutured successfully to an area of sclera that did not show thinning on visualization using 9/0 Prolene suture on a spatulated needle. This was to ensure the plate was secured in place and minimize its movement. The tube was fixed to the Tutoplast^Tm^ to avoid perforating the sclera as the rest of the anterior sclera showed bluish discoloration and thinning. The GDD lumen was occluded via a 3/0 Supramid suture to avoid postoperative hypotony, and the tube was inserted into the anterior chamber through a superotemporal limbal tunneled incision. The free end of the Supramid suture was then tucked to the inferior fornix. Another double layer of pericardial Tutoplast^Tm^ was glued over the tube, and the conjunctiva was placed over the graft (“top layer of patch graft tube sandwich”). Conjunctival closure was achieved via suturing and gluing at the limbus only (Figure 1).

Postoperatively, the intraocular pressure was controlled by full topical intraocular pressure-lowering drops, in addition to systemic acetazolamide, until the Supramid suture was removed 6 weeks after the original surgery to allow for the formation of the fibrous capsule around the tube to avoid hypotony. This was performed by instilling topical anesthesia, making a small conjunctival incision with Westcott scissors at the free edge of the Supramid suture which was placed in the inferior bulbar conjunctiva. Once the free end of the Supramid was exposed, the whole suture was held with Moorfield forceps and pulled out. The patient has completed 11 months of follow-up, her intraocular pressure is controlled at 7 mmHg on no intraocular pressure-lowering medications, her pinhole vision was 6/36 in that eye before posterior subcapsular cataract started to develop, for which the patient underwent a cataract surgery about a year after her tube surgery, and her current best corrected vision is 6/24.

## 3. Discussion

Donor materials including pericardial Tutoplast™ have been used to cover the tube part of glaucoma drainage devices to prevent tube exposure and are performed by most tube surgeons routinely (9).

In this refractory glaucoma patient with thinned sclera who required a glaucoma drainage device to control her intraocular pressure, it was important to strengthen the sclera below the GDD to reduce the risk of scleral perforation.

Other possible solutions previously described for refractory glaucoma patients with thin sclera include strengthening the sclera with other materials such as fascia lata, (10) but in our patient, this would have required another surgical step to obtain fascia lata from the thigh with another surgical wound and longer anesthetic time in an already high risk patient. The use of endocyclophotocoagulation to reduce inflow was described by Rodrigues et al. in a case of scleromalacia and thus avoid the need for more challenging filtration surgery (11). However, we had already performed a micropulse diode laser which had made no impact on IOP control, and a second ciliary body modulating procedure including endocyclophotocoagulation was deemed to have minimal benefit and in our patient especially as they were a high risk phakic patient with abnormal ocular anatomy who had an axial length that was almost 30 mm and a microcornea.

Another solution to avoid the need for scleral reinforcement is to find areas of more normal sclera and thus change the usual supertemporal site of GDD implantation; however, inferior placed tubes carry increased risk of exposure, which may increase the risk of endophthalmitis (12). In particular, our patient had generalized scleral thinning, and there was no advantage to position the GDD elsewhere as scleral reinforcement was required wherever we had placed the GDD.

Our combined “TAG sandwich” technique allowed most of the suturing to be into the Tutoplast^Tm^ avoiding suturing directly into the sclera, ensuring safe plate fixation (13). A two-staged procedure was described earlier by the senior author FA in cases with scleral thinning, to achieve scleral strengthening (5).This novel combined procedure has the advantage of minimizing the number of procedures required under general anesthetic and implanting the GDD immediately for more timely intraocular pressure control.

## 4. Conclusion

We believe our combined “TAG sandwich” technique provides another option in high-risk refractory glaucoma patients with thin sclera or scleromalacia who require filtration surgery. This procedure allowed a safe and effective outcome for our young refractory glaucoma patient with generalized scleral thinning in one surgery, avoiding complications associated with multiple surgeries as well as systemic risks.
